# Sex differences in the prescription of antihypertensive medications in primary care patients: an observational study

**DOI:** 10.3399/BJGPO.2024.0116

**Published:** 2025-07-16

**Authors:** Elisa Dal Canto, Sophie L Theunisse, Michiel L Bots, Frans Rutten, Marion Biermans, N Charlotte Onland-Moret, Wilko Spiering, Birsen Kiliç, Hester M den Ruijter, Monika Hollander

**Affiliations:** 1 Department of General Practice & Nursing Science, Julius Center for Health Sciences and Primary Care, University Medical Center Utrecht, Utrecht University, Utrecht, The Netherlands; 2 Department of Experimental Cardiology, University Medical Center Utrecht, Utrecht University, Utrecht, The Netherlands; 3 Julius Center for Health Sciences and Primary Care, University Medical Center Utrecht, Utrecht University, Utrecht, The Netherlands; 4 Department of Primary and Community Care, Radboud university medical center, Nijmegen, The Netherlands; 5 Department of Vascular Medicine, University Medical Center Utrecht, Utrecht University, Utrecht, The Netherlands

**Keywords:** hypertension, women’s health, primary health care

## Abstract

**Background:**

Pharmacological prescription guidelines for hypertension lack differentiation between the sexes, despite reported sex differences in prevalence, awareness, pathophysiology, and pharmacological response.

**Aim:**

To assess prescription patterns of blood pressure-lowering medication among females and males in primary care.

**Design & setting:**

In this observational study, which was undertaken in the Netherlands, we analysed data collected in 2018 during routine primary care practice among those pharmacologically treated for elevated blood pressure, and free from cardiovascular comorbidities or diabetes mellitus.

**Method:**

We assessed sex differences in the number of prescribed drugs, defined daily dosage, type of antihypertensive medication, and blood pressure control. We adjusted for differences between sexes in age and other covariates.

**Results:**

This study included 8596 females and 5788 males. Both females and males were prescribed on average 1.8 antihypertensive agents per person. Females compared with males were prescribed a significantly lower defined daily dosage (1.8 versus 2.1, *P*<0.001), more often received beta-blockers (35.4% versus 26.3%, *P*<0.001) and diuretics (53.7% versus 50.5%, *P*<0.001), while receiving fewer angiotensin-converting enzyme (ACE) inhibitors (35.4% versus 46.3%, *P*<0.001), and calcium channel blockers (28.5% versus 35.6%, *P*<0.001). No sex differences were found for angiotensin receptor blockers (24.3 versus 24.4%, *P* = 0.842). Importantly, females had significantly better controlled hypertension than males (50.2% versus 45.5%, *P*<0.001).

**Conclusion:**

In those pharmacologically treated for elevated blood pressure, differences between females and males exist in defined daily dosage, type of antihypertensive medication, and blood pressure control, with females achieving better hypertension control than males with different type of medication and lower dosage.

## How this fits in

This study reveals that in primary care, females are prescribed lower doses of antihypertensive medications yet achieve better blood pressure control compared with males. These findings highlight the importance of considering sex-specific factors in hypertension management, suggesting that clinicians may need to tailor treatment strategies more closely to optimise outcomes for both sexes. This research underscores the potential for refining guidelines to better address sex-based differences in hypertension treatment.

## Introduction

Cardiovascular disease (CVD) is a major contributor to morbidity and mortality worldwide, and the prevalence and burden continues to escalate globally,^1^ with hypertension representing a major modifiable risk factor.^2^ According to the World Health Organization (WHO), approximately 1.28 billion adults aged 30–79 years worldwide suffer from hypertension, with only 42% of them being diagnosed and treated.^3^ Hypertension management is far from adequate since four out of five adults with hypertension exhibit uncontrolled blood pressure (BP) levels, exceeding the recommended office values of 140/90 mmHg.^3^ This is of concern as untreated and uncontrolled hypertension considerably increase the risk of future cardiovascular events later in life, independent of other relevant risk factors.^4–6^

Sex disparities in hypertension prevalence and response to pharmacological treatment are influenced by variations in the renin-angiotensin system and immune responses.^7,8^ Females generally exhibit a more anti-inflammatory immune profile than males, potentially impacting BP control.^7,8^ Pharmacokinetic and pharmacodynamic differences further suggest that lower doses of antihypertensive medications may suffice for females.^9,10^

In the Netherlands, where a significant portion of hypertension management takes place in primary care, GPs play a central role in patient treatment. Dutch general practice guidelines recommend a stepwise approach to pharmacological treatment of hypertension, with considerations such as comorbidities, side effects, and personal preference guiding medication choice.^11^ However, these guidelines also do not address potential sex-based differences in treatment strategies.

We set out to investigate the actual prescription pattern of antihypertensive medications, with a specific focus on sex differences. We seek to assess sex differences in the number, prescribed dose defined as defined daily dosage, and type of antihypertensive medication as well as BP control, in females and males with hypertension in Dutch primary care who are prescribed≥1 type of antihypertensive medication, and free from cardiovascular comorbidities or diabetes.

## Method

### Settings and patients

This observational study was conducted using the Julius General Practitioners’ Network (JGPN) database, which encompasses longitudinally routine care of more than 70 general practices serving around 300 000 people in the area of Utrecht, the Netherlands.^12^ Since nearly every citizen in the Netherlands has a GP, except for people experiencing homelessness and those in nursing homes, JGPN provides a representative sample of the Dutch population.^12^ Data are extracted from the electronic health records and each patient–physician consultation is registered according to a systematic format. Reasons for consultation and diagnoses are uniformely coded using the International Classification of Primary Care (ICPC) coding, prescribed medication are coded according to the Anatomical Therapeutic Chemical (ATC) classification standard and hospital referrals are appropirately categorised.

From the JGPN database containing information from 2000–2019, we first selected all patients aged ≥18 years with hypertension (ICPC code K86 and K87). Next, we selected those who in 2018 were prescribed≥1 antihypertensive agent (as the interest was on patients in whom the GP already had prescribed BP-lowering agents). We excluded those with an ICPC code for CVD or diabetes mellitus before 2018, as these patients may be prescribed BP-lowering agents for those conditions rather than for the indication hypertension. Antihypertensive medication used in 2018 was classified according to ATC codes and included diuretics, beta-blockers, calcium channel blockers, renin-angiotensin system inhibitors, and other antihypertensives. Use of medication was only considered if prescribed for>30 days, to exclude other, short-term drug indications.

Patients were excluded when they had at least one of the following conditions: ischaemic heart disease; percutaneous coronary intervention; coronary artery bypass grafting; heart failure; atrial fibrillation or flutter; transient ischaemic attack; stroke; other cerebrovascular disease; peripheral arterial disease; abdominal aortic aneurysm (See **Supplementary Tables 1 and 2** for ATC codes and ICPC codes). This research was conducted in accordance with the European General Data Protection Regulation and within the rules of Dutch legislation.

### Outcomes

Outcomes of interest were the number of prescribed antihypertensive medications, the defined daily dosage, the type of antihypertensive medication, and BP control that were registered between 1 January 2018 and 31 December 2018. Fixed-dose combination pills were counted as two or three distinct medications (1.6% of the study population used combination pills). The antihypertensive medication was classified according to the ATC codes into: angiotensin-converting enzyme (ACE) inhibitors, angiotensin receptor blockers (ARBs), beta-blockers, calcium channel blockers, diuretics, and other antihypertensives. To assess the dosage of antihypertensive medications prescribed for each individual, we used the defined daily dosage methodology. The defined daily dosage is a statistical measure of drug consumption defined by the WHO Collaborating Centre for Drug Statistics Methodology.^13^ It is linked to the ATC code drug classification system for grouping related drugs. The defined daily dosage enables comparison of drug usage between different drugs of the same group, and it was calculated using the following formula for each medication: [dosage * (frequency/day)] / (assumed average maintenance dose per day for the drug given in the literature for each drug). Subsequently, all medication-specific prescribed defined daily dosages were summed to calculate the overall defined daily dosage for each patient.

BP control was assessed using the last recorded office BP measurement from 2018 as documented in the electronic healthcare record. Uncontrolled hypertension was defined as an office systolic BP (SBP)≥140 mmHg and/or a diastolic BP (DBP)≥90 mmHg, the average of two measurements, in line with the Dutch and European guidelines.^14,15^ Patients were grouped as either having controlled hypertension (SBP<140 mmHg and DBP<90 mmHg) or uncontrolled hypertension (SBP≥140 mmHg and/or DBP≥90 mmHg).

### Covariates

Covariates considered in our analysis included the following: cardiovascular risk management (CVRM); history of chronic kidney disease (CKD); dyslipidaemia; and current smoking. History of CVRM was based on the presence of the ICPC code for CVRM, indicating that a patient is enrolled in a primary care CVRM programme for CVD prevention. CKD encompassed patients with the ICPC code for renal disorder or insufficiency. Dyslipidaemia was identified through the ICPC code for lipid disorders or if lipid-modifying agents were prescribed. Smoking status was assessed by reviewing the GP’s EHR for indications of current smoking. Measurements of height and weight were retrieved from diagnostic files of the EHR, and body mass index was calculated as weight (kg) / height (m^2^).

### Data analysis

Characteristics of the study population were presented as means (standard deviation; SD) for continuous variables and as percentages for categorical variables, for females and males seperately. A χ^2^ test was performed to assess whether differences between females and males were statistically significant at a two-sided *P*-value of 0.05. We performed UNIANOVA (Univariate Analysis of Variance) analyses at a 95% confidence interval (CI) and a *P*-value of 0.05 to account for differences in the distribution between females and males. These analyses were adjusted for age, CVRM history, CKD, dyslipidaemia, and smoking status. Analyses were conducted using the Statistical Package for Social Sciences (SPSS; version 26.0).

## Results

Out of the 2018 JGPN population comprising 286 624 people, we identified 14 384 patients (8596 females, 59.8%) with hypertension based on the ICPC code (Table 1). Females were older compared with males (65.2 versus 62.7 years, *P*<0.001), with no significant difference in mean SBP (141.0 versus 141.7 mmHg, *P* = 0.179). Mean DBP was lower in females compared with males (82.4 versus 83.6 mmHg, *P* = 0.031). Additionally, females had more often a history of CKD (7.4% versus 6.5%, *P* = 0.05) and obesity (14.8% versus 9.8%, *P*<0.001), while they were less likely to have dyslipidaemia than males (23.4% versus 27.8%, *P*<0.001) (Table 1).

**Table 1.** Study population characteristics of females and males affected by hypertension, free from cardiovascular disease and diabetes mellitus

	Females (*n* = 8596)	**Males (*n* = 5788**)	*P*-value
Age (years)	65.2 (13.0)	62.7 (12.1)	<0.001
BMI (kg/m^2^)^a^	28.7 (6.4)	28.0 (4.3)	<0.001
SBP (mmHg)^b^	141.0 (16.9)	141.7 (15.8)	0.179
DBP (mmHg)^b^	82.4 (10.2)	83.6 (10.4)	0.031
History of CKD	633 (7.4%)	377 (6.5%)	0.050
History of dyslipidaemia	2012 (23.4%)	1608 (27.8%)	<0.001
History of obesity	1272 (14.8%)	567 (9.8%)	<0.001
History of CVRM	2343 (27.3%)	1621 (28.0%)	0.324
Current smoking	1045 (12.2%)	746 (12.9%)	0.192

^a^Weight and height measurements, or BMI, were not available for all patients, therefore *n* total = 4478 patients, *n* females = 2696, *n* males = 1782. ^b^Blood pressure measurements were not available for all patients, therefore *n* total = 8385 patients, *n* females = 5115, *n* males = 3270. The SBP and DBP indicate the last available BP measurements from 2018 for each patient. Data are presented as mean (SD) or as count (%). BMI = body mass index. CKD = chronic kidney disease. CVRM = cardiovascular risk management. DBP = diastolic blood pressure. SBP = systolic blood pressure. SD = standard deviation

The average number of prescribed antihypertensives for both females and males was 1.8 per day. The distribution of patients receiving one, two, three, or four or more prescribed drugs was 44.5%, 36.0%, 16.0%, and 3.5% in females, and 41.6%, 37.4%, 16.9%, and 4.1% in males. After adjustment for potential confounders, we found no difference between females and males in the number of prescribed antihypertensives (01.8 [95% confidence interval {CI} = 1.8 to 1.8] versus 1.8 [95% CI = 1.8 to 1.9]).

**Figure 1.**
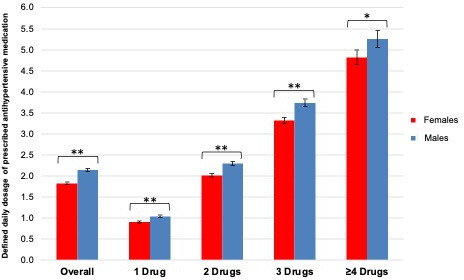
Sex differences in the defined daily dosage of prescribed antihypertensives, overall and in relation to the number of prescribed antihypertensives, based on multivariable analyses. The range of defined daily dosage indicates the 95% CI, and the asterisk indicates a significant difference between females and males (*=statistically significant at a *P*-value of 0.05, **= statistically significant at a *P*-value of<0.001).

Overall, the defined daily dosage prescribed in females was significantly lower than in males (1.8 [95% CI = 1.8 to 1.9] versus 2.1 [95% CI = 2.1 to 2.2], *P*<0.001). This difference persisted across strata of number of prescribed medication (Figure 1 and **Supplementary Table 3**).

**Figure 2.**
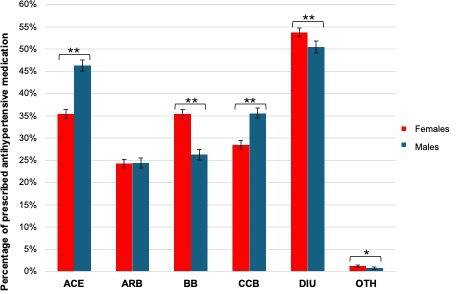
Sex differences in the type of prescribed antihypertensives based on multivariable analyses. ACE = angiotensin-converting enzyme inhibitor. ARB = angiotensin receptor blocker. BB = beta-blocker. CCB = calcium channel blocker. DIU = diuretic. OTH = other antihypertensive. The range of percentages represents the 95% confidence interval, and the asterisk indicates a significant difference between females and males (*=statistically significant at a *P*-value of 0.05, **=statistically significant at a *P*-value of<0.001).

Differences in the types of prescribed antihypertensive medication between females and males were observed (Figure 2 and **Supplementary Table 4**). Figure 2 provides the differences based on multivariable analyses. Compared with males, females were less frequently prescribed ACE inhibitors (p<0.001) and calcium channel blockers (p<0.001), equally often ARBs (p=0.842), significantly more often beta-blockers (p<0.001) and diuretics (p<0.001), and more often other antihypertensives (p=0.002). These findings remained consistent across strata of the number of prescribed antihypertensive medications (**Supplementary Table 4** *)*.

**Figure 3.**
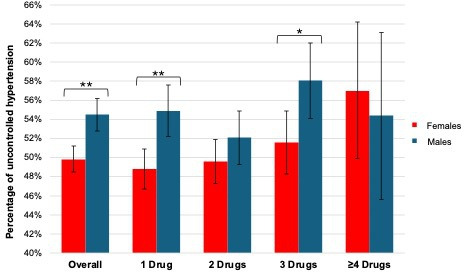
Sex differences in blood pressure control overall, and in the number of prescribed antihypertensives, based on multivariable analyses. The range of percentages represents the 95% confidence interval, and the asterisk indicates a significant difference between females and males (*=statistically significant at a *P*-value of 0.05, **=statistically significant at a *P*-value of<0.001).

BP control was more often achieved in females than in males (54.5% [95% CI = 52.8% to 56.2%] versus 49.8% ([95% CI = 48.5% to 51.2%], *P*<0.001) as shown in Figure 3 and **Supplementary Table 5**. This observation held true for patients receiving up to three prescribed antihypertensives.

## Discussion

### Summary

Our study identified sex differences in antihypertensive prescription patterns. While both sexes received nearly two antihypertensives on average, females were prescribed lower dosages and different types of medications. Importantly, BP control was more effectively achieved in females than males, with 50.2% of females compared with 45.5% of males achieving office BP <140/90 mmHg.

### Strengths and limitations

The study’s strengths include the use of real-life, unselected patient data, reflecting routine primary care^12^ and a large sample size enabling meaningful comparisons. Furthermore, we are among the few studies in the domain of BP in which the dosage of prescribed medication was assessed and compared between females and males. Nonethelsess, certain limitations may have affected some of our findings. First of all, BP measurements were not available for all patients with an ICPC coded hypertension in the year of our study (Table 1). BP measurements take place three to four times a year in those who take part of the primary care CVRM programme, but in others BP is only measured non-systematically or on indication. The availability of the measurements depend on the extractability of the information from the electronic medical records. When a GP does not register the measurements in extrable designated areas in the electronic health care records, the information is not available. Missingness here potentially only affects the analyses on controlled hypertension as these are based on measured levels, not the results on the other outcomes. Given that our findings on controlled hypertension align well with the existing evidence, we assume that missingness did not have a major impact. Additionally, we relied on a single BP measurement per patient for our analyses. While this approach aligns with the most widely used practice in routine clinical care, it may not fully capture variability in BP and could lead to some misclassification.

Another important limitation is the lack of data on medication adherence. While adherence is crucial in determining treatment efficacy.^16^ and could potentially explain some of the observed differences in blood pressure control between males and females, our study was unable to assess adherence owing to the nature of the data available. However, previous studies have indicated that females may be less adherent to chronic medications, including antihypertensives, than males.^17,18^ This suggests that the better blood pressure control observed in females in our study is unlikely to be entirely attributable to higher adherence. Nevertheless, this aspect warrants further investigation.

### Comparison with existing literature

Our findings regarding lower rates of uncontrolled hypertension in females compared with males are consistent with several studies conducted in the past.^19–23^ A recent study from the US analysing National Health and Nutrition Examination Survey data revealed a concerning decline in BP control among adults with hypertension, particularly among women.^22^ Data from 1996–2002 reported that 52% of Dutch women and 68% of men aged 30–59 years had uncontrolled hypertension despite treatment.^19^ A recent US systematic review revealed declining hypertension treatment rates in men, with an increase in uncontrolled hypertension.^23^ Contrary to some previous findings, our recent data suggest a stabilisation in the prevalence of uncontrolled hypertension, with rates remaining alarmingly high but potentially stabilising for females while showing a positive trend for males.

Second, we observed no significant sex differences in the number of prescribed medications, a finding supported by limited evidence from other studies. Two Swedish studies reported that women used fewer antihypertensive medications than men.^24,25^ An older study on US adults found that women used three or more antihypertensives less frequently than men.^26^ However, these studies included patients with CVD and diabetes, complicating direct comparisons owing to varying indications for medication prescription.

Third, we showed that females were prescribed lower defined daily dosage compared with males; a difference that increased with an increasing number of prescribed antihypertensive agents. Better controlled hypertension in females compared with males was achieved by the same number of medications at a lower dosage. This observation finding fuels the discussion on whether treatment guidelines should provide different recommendations in the prescription of type of medication for females and males. Sex-related variations in the renin-angiotensin system and the immune system may contribute to variations in medication efficacy and side effects between sexes.^10,27,28^ Moreover, females have a different pharmacokinetic and pharmacodynamic profile, leading to higher plasma drug concentrations and an increase likelihood of adverse effects.^11,12^ Consequently, lower dosage might be appropriate for females, as demonstrated in recent research on sex differences in heart failure treatment, suggesting that females may require lower dosages of ACE inhibitors, ARBs, and beta-blockers compared with males.^29^ This approach could potentially mitigate side effects and improve adherence, as lower doses may better align with physiological differences between sexes, resulting in improved treatment efficacy and fewer adverse effects. On the other hand, this also raises questions about whether males are undertreated or exhibit lower medication adherence, as adherence plays a pivotal role in achieving treatment goals.^16^ A study analysing pharmacy and medical claims for nearly 30 million adults in the US found that women had lower adherence to chronic medications compared with men, and were also less likely to receive guideline-based drug therapy for conditions such as diabetes and CVD.^17^ Additionally, a systematic review found lower self-reported adherence to antihypertensive therapy in women aged≥65 years.^18^ However, definitive evidence of sex differences in adherence to antihypertensive therapy remains inconclusive, emphasising the urgent need for high-quality studies investigating these issues, particularly among older populations.

Fourth, our study revealed sex-related variations in the pattern of type of medication, aligning with some aspects of previous research.^19–21^ For instance, we observed that females were less likely to be prescribed ACE inhibitors and more likely to receive diuretics than males, consistent with findings from a recent meta-analysis.^30^ However, we also observed a higher prescription rate of beta-blockers in females compared with males, whereas the same meta-analysis reported no sex differences in beta-blocker prescriptions.^30^ This discrepancy may be owing to differences in the populations studied; specifically, we excluded patients with a history of coronary artery disease or heart failure, conditions more common in males and often treated with beta-blockers. Differences in healthcare settings and reimbursement policies across countries may also have influenced these results. Such variations in prescribing practices highlight the importance of considering context when interpreting these findings. Finally, although guidelines suggest that the efficacy of lowering BP is similar among all medication types,^11^ certain combinations may possess higher efficacy and a more favourable side-effect profile. For instance, some studies, reported that ACE inhibitors showed the best BP control rates in men, whereas diuretics were most effective in lowering BP in women.^31^ On the other hand, a higher incidence of adverse effects, predominantly dry cough was reported with ACE inhibitors in women compared with men.^24^ Unfortunately, our analysis lacked systematically reported information on side effects from JGPN database.

### Implications for research and practice

The current study underscores various sex-related disparities in the pharmacological treatment of hypertension within Dutch primary care, particularly at the point when the GP has already initiated antihypertensive medication. These aspects are not addressed in the current CVRM including hypertension management. Our findings emphasise the need for greater awareness and further research into aspects such as side effects, adherence, dosage, and control frequency in this domain. A major implication is that healthcare providers should recognise that females, on average, may need a lower dose of antihypertensive medication than males. This is important when aiming to achieve a controlled BP while minimising side effects.

In conclusion, our study showed disparities in the prescription patterns of antihypertensive medication between females and males. Females and males received, on average the same number of antihypertensives, but differed in the types of drugs prescribed and in the defined daily dosage. Females in our study exhibited better BP control compared with males; however, this observation warrants cautious interpretation given the lack of adherence data and other potential confounders. These findings call for further research and consideration in treatment guidelines to optimise hypertension management while accounting for sex-related differences.

## Data

Data may be obtained upon request from a third party and are not publicly available.
